# Book Reviews

**Published:** 1817-02

**Authors:** 


### CRITICAL ANALYSIS

### *OF RECENT PUBLICATIONS*

IN THE

### DIFFERENT BRANCHES OF PHYSIC, SURGERY, AND MEDICAL PHILOSOPHY.

### *Elements of Pathology and Therapeutics, &c.* By CALEB HILLIER PARRY, M.D.

*(Concluded from our last.)*

BEFORE we enter on the remainder of this performance, we conceive it justice to Dr. Parry to remark the propriety of his commencing a work on pathology with a minute examination of all the forms of inflammation. Most elementary writers, since the time of Boerhaave, have followed the beaten road of attending to fever before any other disease. This, in the then imperfect state of morbid anatomy, was to be expected; but it is impossible to account count for a perseverance in such a mode of instruction, now when we are so frequently informed that the principal danger of fever is inflammation excited in some vital part.

The first division of this work, comprehending the laws of inflammation, in its various forms, and in parts variously constructed, made the subject of our last Number. The rest of the book contains the application of the above to various diseases: the first in order is Dropsy. The reason of this arrangement is, that, as effusion of serum is the simplest form of that increased momentum which constitutes inflammation, so this effusion, in its most simple form, shows itself by anasarcous swellings. These, it is remarked, are often the sequelæ of the former diseased action. Still, however, if they arise from increased action, the author maintains that they are the effect of what is called the phlogistic diathesis. That such a state often exists under high inflammation, we see no more reason to doubt than the author; but whether such is universally the case, will be considered hereafter. That the fluid extravasated in dropsy is also similar to that effused in some kinds of inflammation, admits of no question. The frequent thickening of the respective membranes lining the thoracic and abdominal cavities, we cannot, for a moment, fail to consider as a further proof of the existence of inflammation, whenever such appearances are detected; or that the pain, when it occurs, is an additional proof, strong in proportion to its degree. Even where none of these proofs are present, the author conceives that these swellings may arise from increased momentum, approaching, but not amounting, to inflammation.

Dr. Parry next proceeds to show that this effusion is, for the most part, a salutary action, being the means of lessening the phlogistic diathesis. We agree with him that it is no objection to this opinion if the effusion sometimes proves fatal when determined on the brain; because no new action, however salutary may be its intention, can be entirely divested of danger. All this goes with much regularity, till at last the author, with great candour, introduces us to a condition with which we know not how to connect the idea of increased momentum.

"We may also, (says he,) conformably to the principles above admitted, conceive that easily yielding state of vessels, approaching to that of death, which, in order to the production of effusion, does not require the coincidence of the excessive momentum usually accompanying the phlogistic diathesis.

"It may, however, be repeated, and the reader is requested continually to bear it in mind, as an important practical conclusion, that, in all these cases, the impetus is excessive with regard to the individual individual constitution; and therefore is speedily followed by that process of effusion, which, when in a just degree, it must be nevertheless admitted that nature employs as the most efficacious means of relief.

"If, to the explanation of dropsical effusions which has thus been attempted, it be objected, that accumulations of fluid may arise from diminished absorption, I answer, that, though such a cause is theoretically possible, the question is, whether it be actually true. My observation has not furnished me with a single fact, in direct proof of the agency of this cause; and one could with no shadow of reason attribute it to the many pounds of serum, which are sometimes effused into the abdomen in the short space of twenty-four or thirty-six hours. The arguments in favour of this cause, deduced from the effects of remedies, the operation of which stands equally in need of proof, will not be admitted by any logical pathologist."

If, by the first part of these observations, Dr. Parry means to confine himself to those profuse sweats which sometimes precede death, we shall not dispute whether they arise from a "momentum excessive with regard to the individual constitution;" but, when we know that transudation sometimes follows death, when, as far as we can discover, no momentum exists, we are apt to suppose that in *a moribund* condition it may sometimes exist; and, if this is presuming too much, we must at least admit that these effusions sometimes exist under a degree of debility, and so entirely without pain, that, if excessive momentum exists, it cannot be considered as arising from those causes of inflammation which distinguish the phlogistic diathesis. And, though we readily admit that such rapid accumulations cannot be entirely the effect of a want of absorption, still we are not perfectly satisfied that there is any degree of effusion, however rapid and however considerable, that the absorbents, in a perfectly healthy state, and acting as they usually do, according to the necessity of the part or of the whole, might not take up. We allow, with Dr. Parry, that the effusions into the abdomen, in the short space of twenty-four or thirty-six hours, will sometimes amount to many pounds. But will not the absorption sometimes amount to as many. After a dose of elaterium, do we not often find a quantity of fluid we could scarcely believe voided by the rectum and the urinary organs; and how should this fluid find its way thither but by absorption? and that the effused fluid is absorbed, cannot be questioned, as we find, for a time, that the abdomen is reduced as suddenly as the fluid is voided.

The next subject, arising from increased momentum, which comes under our author's consideration, is Hæmorrhage.

 Here

Here Dr. Parry appears to great advantage, being, we believe, the first person who considered the effusion of blood under the skin, improperly called *purpura*, to be the effect of phlogistic diathesis. Dr. Aikin, in the Memoirs of the Medical Society of London, we believe, first noticed petechiæ sine febre; but, though he proved that this was different from the scorbutic form of that disease, yet he does not seem to have completely decided on its inflammatory character. Dr. Parry very properly remarks, that these spots vary in their colour from a pale red to a purple approaching to black. But this is not the case on their first appearance. If the disease is severe, they appear almost of a bright red at first, and gradually grow paler, or acquire the modena colour. This last arises entirely from the well-known properties of the blood, which, when excluded from air, as it always must be when extravasated within the living body, gradually passes through those changes in its colour. This is very different from the purple spots of the old malignant or spotted fever, or from such as sometimes, but rarely, appear at this time in the early stage of scarlatina or small-pox. In these forlorn cases, the spots will be sometimes of a deep purple at their first appearance, and hæmorrhages from the nose, bladder, rectum, or vagina, will consist of dark-coloured blood, of a creamy consistence, which never coagulates.

A chapter follows on the final causes of Inflammation, Dropsy, and Hæmorrhage. The consideration of this we shall defer for the present.

The author now takes a general view of simple excessive determination or fulness of blood, first as it affects the whole body, and next individual parts. In the former, the determination or fulness is not permanent in any individual part; and, though fulness, and even increased momentum, may exist with wandering pains, yet, as no local inflammation is excited, the patient may be relieved by the abstraction of blood, and the blood may show no inflammatory crust. When, on the contrary, the determination continues for any length of time to a particular part, inflammation is excited. In the cutis its effect is first described with much accuracy, and with a number of good practical remarks: next in the head, the mammæ, the uterus, prostate and thoroid glands, the liver, spleen, and muscles, including those of the heart. In all these the author shows, by many practical proofs, which his genius and industry have enabled him to detect, how frequently the word nervous, and the mistaken ideas associated with it, have warped the practitioner from that attention to lessening plethora, and consequent increased action, which have been the true cause of these complaints.

The

The following, however, is the most remarkable instance of the restorative powers of the economy we remember to have met with.

"Females, whether about the age of puberty, or afterwards, when either chlorotic, or labouring under strong nervous complaints, are apt to suffer great palpitations of the heart on muscular exertions, which, though slight, are considerable relatively to their accustomed habits of indolence. In these cases, the palpitation produced by walking up stairs, or up hill, is accompanied with great uneasiness about the heart, and with very quick and short respiration; and the pulse, even during rest, will sometimes habitually reach from 100 to 120 in a minute. The beating of the heart is usually felt at such a distance from its natural place, and there is often such a difficulty of lying on either side, that one cannot help concluding the heart to have suffered considerable enlargement. In such a case I have known absorption of two of the ribs carried to a considerable extent; and pressure with the finger on the heart through the yielding spot, produced great anxiety, and immediate disposition to syncope. Yet this patient recovered; and now enjoys tolerable health, thirty years after the period of my attendance.

"In other instances, the dissections of strong persons, usually after the middle of life, shew great and permanent enlargements and thickenings of the heart, without apparent inflammation.

"In these cases, as in others before mentioned, there is certainly a tendency to vibrate backwards and forwards between the states of simple fulness and actual inflammation; so that, in the latter state, the blood shall exhibit the usual inflammatory crust. Thus, in the changes which occur in the muscular substance of this important organ, we have another example of increased momentum or fulness, common to the two states of simple excessive determination and inflammation."

In considering the gradations of increased determination or fulness, Dr. Parry enters much into a consideration of the condition of the mucous membranes. In this division, the progress of what we shall take the liberty to call chronic inflammation in the mucous membranes of the nose and pharynx, the bronchia, and intestinal canal, are well marked, with the consequences of that inflammation continuing, or assuming a more acute form; and the frequent sufferings of the patients from a mistaken opinion of the causes of catarrh, dyspnœa, dyspepsia, deafness, ptyalism, &c.

We have next a few remarks on the determination of blood to particular parts, at periods when increased action is necessary, on account of their increased functions.

"The turgescence of the generative organs is an illustration, by a healthy process, of the determination of blood to certain parts, [illegible]nded to be followed by increased secretion or excretion. In rabbits, the skin of the orifice of the vagina becomes almost black, by extreme vascularity, during the season of heat; and in rams, at the usual rutting time, not only the testicles enlarge, but the skin about the inside of the thighs becomes of a florid redness, Castration, at this period, is almost uniformly followed by inflammation and death.

"The periods of menstruation in many females are preceded by pains about the back and loins, accompanied by a sense of great weight. These pains, which are owing to a great degree of turgescence in the hypogastric arteries and their ramifications, usually cease as soon as the natural discharge is fully established. This turgescence, and the gradual decrease from the sanguineous evacuation, seem to be demonstrated from the fact stated above."

This chapter concludes with some general remarks on ephemeral fevers arising from occasional fullness, which are relieved by copious perspiration. These processes, the author has observed, are not always extended over the whole surface, but often confined to individual members.

These considerations necessarily lead the author to the doctrine of Stimuli and Sympathies, as the only means by which impressions can be excited in various parts, or on the whole machine, so as to induce them to set up new action. To explain this, as far as it can be detected, a knowledge of the structure and functions of the nervous system is required, and the epitome given by our author is alone enough to give currency to his work. Considered as an epitome, our readers will not expect any attempt to shorten it. We shall, therefore, content ourselves with a few extracts, connecting them together as well as the nature of the subject will admit.

"Conformably (says Dr. Parry) to the true mode of philosophizing, we must begin with considering those animals which are most simple as to their faculties and structure, and thence extend our researches upwards to those which are more complicated and noble.

"In the confervæ, there is no trace whatever of any alimentary canal. In the polypi, and many others, the greater part of the body is a tube, adapted for the reception of food. The substance, of which these animals are composed, is an uniform, pulpy, gelatinous, and somewhat granulated mass.

"In the classes of animals next above them, in which there is the superaddition of sanguiferous vessels, the gelatinous mass is divided into knots; from each of which arise evident nervous filaments, going to different parts of the body, to which the number of these masses or knots is proportioned.

"MM. Gall and Spurzheim consider these knots as true ganglia, each of them, with its out-springing filaments, constituting a distinct nervous system; and the several nervous systems of the viscera of these inferior animals, as the types of those which should should be found in animals of higher rank, having similar parts with similar functions.

"Such systems actually exist, and are those of the plexuses of the thorax and abdomen, and of the ganglia of the Great Sympathetic or Intercostal nerve.

"The existence of these systems in animals, which have neither brain nor spinal marrow, clearly demonstrates that they do not originate from either of those parts; but that they are independent systems, derived from their own gelatinous masses, and each having its own peculiar functions; although, conformably to the opinion of various physiologists, and more especially Bichât, these ganglia communicate by nervous filaments with each other, with the spinal marrow, and with the brain.

"In reality, in proportion to the rank which created beings are intended to hold in the scale of existence, and the additional functions which are suited to that gradation, new parts are added; which, for the purposes of minutely varied and reciprocal influence, require numerous communications between each other and the whole.

"On this principle, we can easily understand why the system of ganglia of the great sympathetic nerve in the thorax should have its origins and functions distinct from those of that in the abdomen; and why each should have its several communications, by nervous filaments, between its own several parts, as well as with those in the abdomen, in the spinal marrow and brain.

"Ganglia and plexuses, though they differ in size, form, and colour, are every where penetrated with blood-vessels, and contain gelatinous matter, which may be considered as being of the same nature with the cineritious or grey substance, abundantly existing in other parts of the nervous system. All swellings, containing nerves and grey substance, are ganglia.

"As, wherever white nervous filaments exist, whether in what is called nerve, spinal marrow, medulla oblongata, cerebellum, or cerebrum, grey substance is found at their origin, MM. Gall and Spurzheim conclude, that these filaments are generated by it; whence they denominate it the Matrix of the nerves.

"Conformably to this view, it will be found, that white filaments not only originate from grey substance, but, wherever they pass through or come in contact with it, receive from it additional filaments; in consequence of which their bundles increase in size; so that nerves may be considered as virtual cones, of which the apices are towards the first origins."

We confess ourselves not perfectly reconciled to these terms, *origin* and *Matrix*. The first, though it may be sanctioned by its application to muscles, where it means only one extremity, yet, as there was a time when it was thought that the nerves *derived* their *origin* from the brain, we could wish it was now discontinued. On this subject we have before offered our opinion. The term matrix is still more objectionable; jectionable, as it has a poetical air; yet we admit it would be difficult to find a better term, or, perhaps, any other single word whatever for a part whose office seems to be to give nourishment to the nerves.

"Each side of the spinal marrow is considered as made up of a succession of continuous ganglia, consisting of grey substance, which exists within a regularly disposed form, and from knots or swellings in which, visible throughout its whole length, in all animals in which this part exists, arise nervous filaments. At each of these swellings the filaments emerge, above and below, or before and behind, relatively to the natural position of the animal; and equally on the internal and external face of each swelling.

"These swellings, or ganglia, are greatest where the nerves arising from them, conformably to the functions of the parts which they are to supply, are largest."

From the peculiar property of those nerves to which we attribute the organs of the senses, and the well-known parts of the brain with which they are connected, and from this some other arguments, Dr. Parry seems disposed to admit, without entering into the discussion, that certain parts of the brain may have certain properties peculiar to themselves. At all events, he pays that compliment to genius, industry, and fidelity, which, we doubt not, must hereafter be echoed in other quarters.

"This short abstract will serve to give as general a notion of the anatomical view of the nervous system, by MM. Gall and Spurzheim, as is consistent with the object of this work.*"

In considering those motions which are deemed involuntary, the author shows that we have no certainty that a nervous influence is necessary; still less than any communication with the brain is required. It is true, in the various complication of ganglia and nerves, that almost every part will sympathize with another; but this is found in animals that have no brain nor nerves. Even in man,

"That the communication by nerves is unnecessary to motion connected with sensation, is evident in the case of the iris; which does not move by any known stimulus applied to its own fibres,

---

"* Those, however, who would study it in the detail which it deserves, must laboriously dissect from the very accurate plates of those authors, accompanying the first volume of their 'Anatomie et Physiologie du Système Nerveux en général, et du Cerveau en particulier;' after having carefully perused the descriptions and reasonings in that work, and in another, by the same authors, entitled 'Recherches sur le Système Nerveux en général, et sur celui du Cerveau en particulier;' both of which may be considered as models of profound investigation, and luminous exposition."

but but suffers elongation by the influence of light, and, I believe, light only, on the retina, with which it has no nervous communication.

"On the other hand, that the intervention of the brain is not necessary to the association of certain muscular movements from slight irritation on a distant part, such as usually produces sensation, is evident from the case of a fœtus, in which there was not the smallest vestige of a cerebrum or cerebellum, but which, even twenty hours after birth, moved up its knees, when the soles of its feet were tickled, sucked my finger, when introduced into its mouth, and a few hours before, had passed fæces and urine, and swallowed food. It is true, that, in this case, there were a spinal marrow and medulla oblongata; but the same motions are recorded as having occurred in another example, in which both these parts, as well as the brain, were totally wanting. From the great resemblance, in other respects, in all the cases of this kind, there is no reason to doubt, that the relation of this fact was strictly true."

This chapter concludes with some remarks on Sympathies and on Automatic or involuntary Motion.

The mental faculties are next considered, first, as connected with the senses; and, next, as both are influenced by the structure and peculiar condition of various organs. After dismissing, with the levity to which, in our opinion, it ought to be consigned, the fanciful lengths to which the science of temperaments, as it is considered by some, have led some modern philosophers, the names of Gall and Spurzheim are introduced with becoming modesty.

"Of late (says Dr. Parry) the physiologists whom I have before quoted, have viewed the subject in another light; and have endeavoured to show, that the capacity for the respective arts and sciences, as well as the sentiments, moral tendencies, and other intellectual faculties, are connected with the comparative proportions of certain parts of the convolutions of the brain, and indicated by the proportions of corresponding parts of the cranium.

"These conclusions, which are illustrated by those physiologists with great force of demonstration, have not hitherto received the sanction of general experience. But to deride them solely on that account would be highly absurd; since we are justified in concluding, that, as it has pleased Providence to make an organized material substance the medium of all the mental faculties, these several faculties may depend, for their existence, on certain parts of the organized mass, and for their degree, on the proportion of those parts."

The consideration of the brain and nerves, naturally introduces us to what is called "the Nervous Constitution," which Dr. Parry attributes principally to a too great determination of blood to the brain, or to certain nerves. In this chapter are included all that variety of complaints so generally, but, we must admit, indolently, consigned to the common term of nervous; vertigo, spasmodic asthma, hiccup, noises in the head, pains in the same; epilepsy, to which several pages are devoted, abounding with useful remarks; convulsions, subsultus, muscular twitchings, tremors, chorea, stammering, shaking palsy, wry neck, tetanus, rabies, sopor, hysteria, hypochondriasis, insanity, dreaming, febrile delirium. All these are attributed with only a few qualifications to excessive impetus of the blood to the brain. The author admits the proof is not unattended with difficulties, and that, in some instances after death, no particular fulness of the vessels, or other marks of this increased impetus, can be detected; but this, he conceives, may arise from the confined locality of the increased momentum, the temporary nature of the paroxysms, and from the increased impetus having ceased in *articulo mortis*. The enumeration is continued to catalepsy, hydrocephalus, hæmeplegia, apoplexy.

The same condition of the spinal marrow or nerves, induces, in the author's opinion, paraplegia, or paralysis of the lower limbs, when unconnected with mechanical pressure; sciatica, affections of the ulnar nerve, tic douleureux. As this last concludes the chapter, we shall transcribe the passage.

"To this head, also, (says our author,) may, probably, be referred that painful disorder called Tic Douleureux; which seems to occupy the extreme ramifications either of the facial nerve, or of the second or superior maxillary branch of the trigeminus. All the circumstances induce me to attribute this pain to increased vascularity, or determination of blood (perhaps amounting to inflammation) to the neurilema, or vascular membranous envelope of those nerves. I form this judgment, first, from the strong analogy which the case itself bears to those before mentioned, in some of which dissection has demonstrated the cause: Secondly, from the extension of the disorder to the branches of more than one nerve in the same patient, which can scarcely be produced, but through the medium of common blood-vessels; since there is no evidence to prove the extension of pain, by pure sympathy, to anastomosing branches of nerves derived from different trunks: Thirdly, from the disposition in this pain to be increased or diminished by those means, which increase or diminish the motion of the heart: And, lastly, from the resemblance of curative effect produced on it, in common with those diseases, which evidently arise from excessive sanguineous determination, by certain remedies, such as abstraction of heat, eau medicinale, and arsenic.

"To me it seems, that this conclusion is much supported by the result of the operation performed by Dr. Haighton, although a different inference is drawn from it by that acute physiologist. The circumstances, which he relates, rather prove the division of the arterial branch supplying the affected ramification of the trigeminus minus nerve, than the division of that ramification itself. This conclusion is farther strengthened by the fact, which has occurred in some other cases, of a return of pain after the functions of the divided nerve have been restored by a regeneration of its substance; since it will readily be allowed, that, as the new nervous formation is the result of a certain action of blood vessels, or capillaries, the morbid state must be produced by the same agents."

As an additional proof of one common origin of diseases, viz. the increased momentum of the blood, the author instances the peculiarities of certain families, the individuals of which were liable to disease in different parts of the same organ, or in different organs, but all of them evidently arising from this cause.

"Thus, (says he,) with regard to the head, I have known one person maniacal, a paternal cousin hæmorrhagic and epileptic, and almost all his children subject either to epilepsy, head-ach, bleeding at the nose, or hydrocephalus.

"In another family, the mother was epileptic, a son laboured under long and excruciating head-achs, and a daughter died of hydrocephalus.

"Of two sisters, one was liable to eruptions on the face, the other to flushing heat of the whole head, and violent nervous affections, without eruption.

"Of two other sisters, one died at adult age of hydrocephalus; and the other had a florid complexion, was greatly disposed to head-ach and hysteria, and had suffered a violent attack of erysipelas of the face and head.

"In another family, a young female had head-achs, great flushing in her face, and bleeding at the nose; her sister was highly nervous and irritable, and two brothers were maniacal."

This argument is further strengthened by what the author calls, "Relation of Diseases by extension," and by remote changes: these constituting the contiguous and remote sympathy of Mr. Hunter, and by relation of diseases by conversion, where the sympathy is dissimilar from the mode of action in the parts under disease. These conversions, the author shows by several instances, are sometimes spontaneous, at others the effect of remedies.

This chapter concludes with the following summary.

"It will have been observed, that the changes above related, include the following maladies; which, exclusively of those arising from the influence of mechanical causes, or of infectious or other miasmata, are the principal to which the animal frame is liable.

"First, excessive determination or momentum of blood to the skin.

"Sweating, scarlatina, measles, erythema, erysipelas, and all the forms of eruptive diseases.

" Secondly, to mucous membranes.

" Coryza, catarrh, whooping cough, croup, sore-throat, peripneumonia notha, catarrhus senilis, bronchitis, asthma; aphthæ, dyspepsia, diarrhœa; and various other disorders of the villous coat of the alimentary canal; strictures in the urethra, œsophagus, colon, and rectum; gleet; fluor albus; catarrhus vesicæ.

" Thirdly, to serous membranes.

" Phlegmon; pleurisy; pericarditis; peritonitis of different parts, constituting enteritis, puerperal fever, &c.; inflammation of the tunica vaginalis testis.—To synovial membranes, producing arthritis; together with the effects of these several states, anasarca, hydrothorax, hydropericardium, ascites, hydrocele, effusions into joints, adhesion, anchylosis, &c. &c.

" Fourthly, to various other membranes.

" Of the spinal marrow or nerves, paraplegia, sciatica, tic douloureux, &c.—To the epithelion, deafness.

" Fifthly, to glandular parts.

" Cynanche parotidæa, or mumps; swelling and other disorders of the thyroid gland, mammæ, testicles, prostate, and various other glandular parts; phthisis pulmonalis; atrophy.

" Sixthly, To the head.

" Head-ach, vertigo, sleeplessness, common nervous affections, mania, delirium, convulsions, hysteria, epilepsy, catalepsy, inflammation of the pia mater, or arachnoides; together with their occasional sequelæ, hemiplegia, apoplexy, hydrocephalus, and other effusions.

" Seventhly, to other parts in various forms.

" Peripneumony, enlargement of the heart, liver, spleen, kidnies, testicles, and uterus, with or without inflammation; fungus hœmatodes, ophthalmia, cataract, amaurosis.

" Eighthly, various increased natural discharges, not already specified.

" Ptyalism, diabetes, lachrymatio.

" Ninthly, morbid depositions, not above arranged.

" Scirrhosities, indurations, ossifications, chalk-stone, biliary and renal calculi, and other hard deposits in different parts.

" Tenthly, hæmorrhages.

" From serous, mucous, or other membranes, or parenchyma; as from the nose, uvula, fauces, lungs, stomach, intestines, kidnies, bladder, uterus, vasa deferentia, skin, liver, &c. To which may be added, the various forms of purpura.

" Such are the chief examples, which occur to me, of diseases, in which increased impetus, momentum; or determination of blood, forms one essential link in the series of previous phenomena or causes.

" In what particular cases they depend merely on excessive local momentum, and in what there is the coincidence of increased action of the heart, it may be difficult, in every instance, accurately to decide. The same symptoms may arise under both states; but, as inflammation, hæmorrhage, and dropsy, may occur without excessive action of the heart, and yet usually exist in the greatest degree, when that action is greatest; so, probably, cæteris paribus, the same may be asserted under the condition of increased local impetus, without inflammation. Farther light will be thrown on this part of the subject, when we come to the more particular consideration of the phenomena of the several diseases arising from this general cause."

A short chapter follows on the "Defective Determination of the Blood." The consequent diseases are, as may be supposed, few; they are, however, sufficiently marked.

Some remarks succeed on the "salutary processes." This part is less connected, and, in some instances, to us very unsatisfactory. Indeed, it has ever been our wish, to avoid as much as possible a too early recourse to final causes. On this account, when our author prepared us for some remarks on the shivering previous to a hot fit in ague, or symptomatic fever, we expected some suggestions concerning the cause of the cold which induced that shivering: instead of this, we have explained to us, that this muscular motion is a means of propelling the blood through the veins to the heart, where it is suggested that there may be an accumulation of heat. The struggling in hysteria, the convulsions in epilepsy, are explained in a somewhat similar manner. The whole of this chapter is more unconnected and miscellaneous than any other part of the work, which is easily accounted for, as there is no difficulty in proving that most diseased actions arise from attempts in the constitution or parts to restore themselves. But we should be careful how we extend this inquiry still further, and admit, with the author, that the advantage of civilization, by procuring an easy mode of subsistence, is a constant cause of inducing plethora, which must be returned by some means or other. Now it is certain, that those whose diet is the most simple, and whose labour the greatest, are often the subject of the most violent forms of inflammation. In the midst of all this, we meet with a very just remark on another order of pathologists, who refer every thing to the digestive organs.

"The particular diseases (says Dr. Parry) which are most apt to occur from the operation of the primary causes, without the concurrence of any observable specific causes of excitement, are, especially, those of the head and alimentary canal.

"These two maladies are often totally independent of each other; so that various determinations of blood to the head, usually called nervous, occur in thousands of instances, in which all the phenomena, constituting the digestive functions, are perceived to be in their just degree and order; and, on the other hand, various for[illegible] of dyspepsia often exist, without the least disorder whatever of the head."

But,

But, whilst we perfectly agree in the absurdity of imputing all diseases to one cause, we wish Dr. P. to be careful of imputing too much to the momentum of the blood. We mention this, because this chapter, the whole of which we have read with great satisfaction, contains so many good things that we are perpetually wishing they had been differently disposed. The greater part would make a most excellent string of aphorisms, but, considered as arguments and inductions, we have found many parts unsatisfactory.

A few remarks, as they appear to us, somewhat misplaced as well as unnecessary, follow, on the excessive direct determination of blood from cold; after which we have the following recapitulation.

"First, that the far greater number of the diseases incidental to the human frame, depends, at some point or other of that succession of antecedent circumstances, which constitutes the chain of causes, on excessive momentum of blood, whether local or general.

"Secondly, that this momentum is not, necessarily, always excessive absolutely, that is, in relation to the usual state of perfect health in the mass of mankind; but relatively to the state of the individual at the period given.

"Thirdly, that many of those movements, which constitute what is called disease, and which, for the time, produce disorder of the different functions, whether of body or mind, are, in reality, processes, the general tendency of which is to restore health, and to prolong life; although, on particular occasions, their operation may be either defective on one hand, or excessive on the other; or may be even sometimes directed to parts, which seem, as it were, unnecessarily implicated in the vain and fatal conflict.

"The view which I have thus taken of animal pathology, is consonant to that simplicity which pervades all the other known operations of nature. The blood is the material from which, by the aid of its appropriate system of vessels, the animal is formed, its life preserved, and its health maintained; and by the immediate affection of the same system, it chiefly suffers disease, decay, and death.

"This theory is of a very different character from those which deduce the greater number of diseases from an assumed disorder of one particular viscus, or local function. It is founded on an observation of certain like phenomena, occurring in a system existing in every part of the body; and, therefore, constituting a law, under which are comprehended the affections, not of one part only, but of the whole frame.

"Neither is it at all incompatible with the opinions of certain ingenious pathologists, who would investigate the ulterior design of the several morbid movements of the animal system. On the contrary, while it admits, and even assumes, the general principle of of salutary purposes, it tends to ascertain the means, or instruments, by which those purposes are effected.

The facts, by which the law has been illustrated or proved, have been, with a few specified exceptions, derived from my own experience. The results only, and not the particulars, have, for the most part, been given.

"For the pathological opinions, I am solely responsible, except where the obligation has been expressly acknowledged; and even with regard to the anatomy, little has been admitted which I have not myself verified by repeated dissections. The process, which I have followed, has been strictly conformable to the principles of science expressed in the commencement of this work.

"Various repetitions will have been observed; but where it is designed to establish the order, as well as the quality, of phenomena, such repetitions are absolutely unavoidable; since the same fact, or principle, must often necessarily form the nexus, or connecting link, of different series.

"What degree of accuracy there may be either in the notation or arrangement of the phenomena themselves, and, therefore, what truth in the theories, it will be for others finally to judge. No fact, unassigned to some other observer, has been admitted, which I have not myself witnessed: and since the operations of nature are as uniform as they are simple, it is probable that he, who, with adequate opportunity, candidly and diligently seeks for truth, will not be wholly disappointed in the pursuit.

"It still remains for me to show, how far the principles, which I have endeavoured to establish, accord with the causes and symptoms of the several diseases, on which I profess to treat; and, more particularly, with the most successful means of accomplishing their prevention and cure."

Such is the best epitome our limits would permit us to make of this truly ingenious performance. It cannot be expected we should do complete justice to such a work, nor that we should give our opinions sereatim on every part. We are further prevented from this, because, till our author enters more at large on the therapeutic part, which we are taught to expect in another volume, his whole system is not fairly developed. We shall, therefore, content ourselves with some general remarks of our own on the principal topics contained in the work, as far as it is given to the public.

That inflammation, in its various stages, in parts variously formed, is the most common cause of disease, we have no scruple in admitting, nor that inflammation is usually attended with encreased momentum in the blood. Equally ready are we to admit, that plethora is the most common cause of spontaneous inflammation, that is, of inflammation without external injury. We admit too, that what is found to to be plethora under a change of action, in a part or the whole system, may have existed with little inconvenience as long as the common healthy actions of life are maintained. But, when a new action of any kind is excited, the increased momentum of the blood, and its determination to certain parts, becomes a source of disease, and the most common form of disease is either hæmorrhage or inflammation. About the age of puberty, when a large quantity of blood is necessary for the growth of the animal, if the quantity of blood is greater than the growth requires, hæmorrhage is very frequent: and provision is made for the external passage of blood by the number of superficial arteries in the nose and about the rectum. In more mature life, if plethora occurs from other causes, the uterus in females, and, in still more advanced life, the urinary organs in men, are the most common outlets.

Notwithstanding all these provisions, plethora will be sometimes determined to other parts, and the consequence will sometimes be hæmorrhage, where no provision is made for an outlet. The most serious of these is the head, and here the greatest provision is made by the relief from the nose, or, if that does not take place, by the quantity of venous compared with the arterial capacity in the brain and neighbouring parts. If all the vessels are overcharged, sometimes hæmorrhage and consequently apoplexy follows; but, more frequently, especially in early life, inflammation takes places. By this process, all the vessels, even the most minute, are strengthened by the effusion of coagulated lymph, as is evident by the increased firmness of the brain, as well as its increased vascularity always discoverable after death from these causes.

If, as is often the case, the determination is to the membranes of the brain, or any other membranes which line what are called cavities, these parts also are strengthened by the effusion of lymph which, uniting the membrane to the part in contact with it, prevents what Dr. Parry called the "relation of disease by extension." Such is the more usual, and may be called the healthy, mode of action in these parts, and is one form of Mr. Hunter's adhesive inflammation. But, if serous effusions are induced by inflammation, the form is changed to the erysipelatous, and there are no bounds made to the extension, but the limits of the membrane itself.

In mucous membranes, the first effect of inflammation is an increased secretion, which approaches nearer to serum in proportion as the inflammation increases. If it goes beyond that form, it approaches almost to the adhesive inflammation of the membranes which line the viscera. Coagulated lymph is is thrown out, which adheres to the mucus surface, but does not unite the sides. Of this kind is the membrane effused in croup, the slough in scarlatina, caruncles in the urethra, and those effusions in the worst kinds of dysentery, which end in sloughs and consequent ulceration.

In this hasty view, we have noticed only the more violent forms of inflammation. The chronic, which, as far as we understand Dr. Parry, he conceives the most common causes of nervous diseases, are much too complicated for us to enter on. We are, however, ready to concede all that Dr. Parry requires on this head, and only wait for his next volume to instruct us in the mode of treatment, which must, at the same time, introduce a more minute detail of symptoms and their probable causes.

---

*An Essay on the common Cause and Prevention of Hepatitis, or Disorder of the Liver, and of Bilious Complaints in general, as well in India as in Europe; with an Appendix particularly addressed to the Medical Profession, recommending the Old Submuriates of Mercury in Preference to those now in use.* By CHARLES GRIFFITH, M.D. Deputy Inspector of Hospitals, and late Senior Surgeon to the Forces.—London: Highley and Son. 8vo. pp. 202.

WE know not why a surgeon should write on medical subjects with more apparent levity than a physician; but, certainly, there is a sportiveness throughout this essay, which does not perfectly accord with our grave notions of things, and almost induces us to remind the author, that, though surgeon to the forces, he is also M.D. Nor have we found quite novelty enough to authorize the publication of a book. The hints, numerous and useful as they are, might have been compressed into a few sheets in some compilation where, perhaps, they would be more generally read than in their present form.

The remarks on diet and clothing are such as have been urged very often, though we admit that we have met with some useful cautions on both these points. We cannot, however, quite reconcile ourselves to the urgent and indiscriminate recommending of exercise in warm climates, nor to considering the want of it as the most common cause of hepatitis. We shall offer a short extract on this subject, to show that the force of argument is not overlooked by the author, though it may be obscured to the reader by the unnecessary introduction of extraneous matter.

"Having fairly, however briefly, contrasted the habits of the Company's civil servants in the East with the Europeans of the West

West India Isles, I must be allowed in support of the justice of my observations regarding the cause of this disease particularly in the East, to revert here to duties performed by a very numerous body of Europeans in the service of the India Company, with an exemption from this malady, equalling what is enjoyed in Jamaica or Barbadoes, the Company's military establishment will immediately occur to the reader, and afford to the unprejudiced mind further conviction of the cause of such exemption, and that this obstruction is not brought on by miasmata, but by a species of indolence, which exceeding indeed any I have witnessed elsewhere, I am willing to call *Indigenous Indolence.*

"Soldiers, it will be recollected, are fortunately compelled by the nature of their duties to make use of their own limbs, and as it would be rather awkward to scale a wall or storm a battery in palanquins, so a charge of bayonets, whilst carrying umbrellas over their heads, might prove inconvenient; nor, although they allow the assistance of their numerous followers in other matters, will our obstinate countrymen, when an enemy presents himself, consent to fight by deputy: thus then, whilst encamped, they do a number of things of necessity, and the consequence is, that, whilst they keep the field, recent bilious complaints are little known amongst them; but no sooner do these men return to garrison duty, and a state of relaxation, than they commence a participation with civilians in the fruits of indolence, although in a less degree; with this fact the surgeons, both of the line and in the Company's service, are so well acquainted, that a further reference becomes unnecessary.

"To adduce further proofs of the most common origin of inflammations of the liver, would, I think, be superfluous; and if some specious physiologist, who may have been long looking towards this monster in the moon, should be unwilling to yield his assent to a truth so simple as that I have stated, he will fall only into a common error without altering that truth.

"In all matters not quite self-evident, we are too apt to sally forth armed at all points for their encounter, equipped with our most formidable apparatus, and being determined to exhibit our profundity at least, we angle for the desideratum, as the learned Johnston did whilst deriving his monosyllables, i. e. with a five-fathom line, so that, passing the fry we seek, we plunge into deep water, whereas the subject of our enquiry, as Mr. Horne Tooke exhibited respecting the above, in his 'Diversions of Purley,' lies often within a foot of the surface. Many far-fetched theories have thus been invented for elucidating the cause of hepatitis, or the liver complaint of India; but it has fared with the most laboured of these, as it commonly fares with very complicated mechanical inventions, that they are too ingenious to be of practical utility."

The appendix being principally a collection of facts to show the advantage of the old mode of making calomel by sublimation six times, and also of other submuriates of mercury, cannot well be epitomized. There is, however, a very useful useful hint at the conclusion, which we shall extract for the benefit of our readers, especially as the experiment may be tried without injury to their patients.

"I could have adduced (says Dr. Griffith) an abundance of cases illustrative of the superiority of the old submuriates, and, in my judgment, perfectly conclusive, but I decline this, for the following reasons: if an author's assertions command credit, he wants not such addition; if they do not, cases will add little to their authenticity, although much to the size of his book, and this latter consideration alone is sufficiently weighty. And now, having offered all I proposed to point out in the foregoing pages, i. e. a more manageable tool than what is in use, and one that will forward a tolerable workman's intention with sufficient dispatch without cutting his fingers, I have no apology for detaining the reader further than whilst I make one practical exception to the general rule laid down concerning the deobstruent operation of mercury on the whole glandular system; and this applies to the enlargement of the spleen often accompanying, and often consequent to, remittent and intermittent fevers, in India called the Boss, in England Ague Cake. In this obstruction, I must confess, mercury, contrary to the above rule, is so insignificant, that I have sometimes on that account been almost inclined to doubt the glandular construction of this viscus, all other glands evincing its deobstruent power.

"Being first disappointed in two cases which occurred to me of this description at Calcutta, in each of which I had twice excited the salivary glands to no purpose, the enlargement still increasing, I was urged by the dread of suppuration to make trial of the succus conii inspissatus, which I persisted in, having luckily preserved some in my own little stores, until I had repeatedly affected the head, the tumor in both cases gradually lessening, until at length it was wholly done away. Had the above been insufficient to establish the good effects of the conium, the expedition to Walcheren (during and subsequent to which this disease prevailed but too fatally) afforded me ample proof of its efficacy."

We are ready to admit our frequent, perhaps we might say constant, disappointment in attempting to remove these enlargements by mercury; for, we very much suspect, that, in the instances in which success has followed this treatment, the tumors would have subsided, as we have found them do, without any remedies whatever. We are, therefore, very glad to be instructed in any mode of proceeding.

---

*An Essay on the Gripes of Horses*; by Bracy Clarke, F. L. S. and Natur. cur. Berolin.—8vo. Sold by the Author, London.

We have the happiness to live in an age, when this noble and truly valuable animal is rescued from the fangs of the ignorant ignorant and mere empiric. But this was not the only disadvantage attending its former condition. Many of the farrier's remedies were, doubtless, successful; yet, as long as mystery was preserved by them, the science never could be progressive.

The most serious, because the most rapid of all the diseases to which horses are liable, is gripes. In this little tract, Mr. Clarke shows, in a most interesting manner, the difficulties he for a long time encountered, in his attempts to arrive at the cause of sudden fatality. At length, a few dissections have given him complete information, and directed him in a mode of treatment, which, if attended to early enough, has not failed in a single instance. The remedy is easily prepared, but our medical readers will see the impropriety of offering it to those who are not competent to judge of the disease, and also of the causes which may interfere with the success of the remedy. We can, therefore, only recommend the perusal of this valuable paper to all such as undertake the veterinary art, either by itself, or with any other department of medicine.

---

*Observations on the projected Bill for restricting the Practice of Surgery and Midwifery to Members of the Royal Colleges of London, Edinburgh, and Dublin; and to Army and Navy Surgeons: with some Modifications proposed, by which the Measure will be more compatible with the true Interests of the Public; and not oppressive to the present Race of Pupils for the Profession.* By a GENERAL PRACTITIONER. —Bent, London. Pp. 31.

"Leave having been obtained towards the close of the last session of parliament, for the introduction of a Bill 'To regulate the Practice of Surgery and Midwifery throughout the United Kingdom of Great Britain and Ireland,' the proposed measure becomes a fit subject for discussion—bringing it fully and fairly before the public, if it has intrinsic merit, will be useful, and ensure it public support—and without intrinsic merit and public support, a measure so important, involving so materially *public health* and *individual rights*, cannot be expected to receive the sanction of an enlightened legislature, which assents only to wholesome laws for the good of the nation."

In considering these questions, the author divides his subject into the three following heads.

"First, the amount of what the Bill denominates, 'the usual fee' for the diploma, which may be demanded and received; and the qualification required for such diploma.

"Secondly, whether, while the public good requires that students of proved attainments only be in future allowed to commence practice in surgery and midwifery, the actual wants of the public will allow of such a diminution of educated professional aid, as may be expected to result from the exaction of the increased expenses and fees for obtaining the college diploma.

"Thirdly, whether it is just that the present race of students who have studied according to the late regulations of the Royal College, and who are equal to the test of a rigid examination, but whose pecuniary means are not equal to the increased hospital attendance and expenses, shall be excluded from two branches of a profession for which they have been regularly educated.

"As to the first question, 'the usual fee' for a diploma from the Royal College of Surgeons in London, is now thirty-two pounds ten shillings for a practitioner in or within seven miles of London, and twenty-two pounds for one beyond that distance;—to which amount, it was increased the year before last; when it was also regulated, that every candidate for examination should have attended an hospital for *one year*, instead of *six months* as formerly.

"In respect to the second question, it may be observed, that while the diploma of the Royal College remains, as at present, an honorary appendage only to the professional character (for it is not only unnecessary for country practice, but even in London a profession of *Surgery* adorns the showy fronts of the retail apothecaries' and chemists' shops uncontrouled by the College) no objection can be made to whatever may be the preliminary expense for obtaining it;—but, when the petitioners for this Bill are seriously asking parliament to enact, that the diplomatist of a Royal College, and an army or a navy surgeon only, shall, even in the most remote parts of the kingdom, or under any circumstances, afford surgical relief in bodily accidents, where the rapid strides of inflammation, if not arrested, may be fatal—or give scientific assistance in difficult emergencies in midwifery, which only may be able to save life, the expenses of such diploma become of the first importance, and call for investigation. In many districts, it seems that no regularly educated professional man is now resident, within ten, twelve, eighteen, or even sometimes twenty miles; and, yet accidents occasionally occur—a scull is fractured—a limb is dislocated—an artery is wounded—and there may be the most urgent occasion for competent professional aid.

"To the public, then, the proposed measure must be injurious, if it unnecessarily augments the expenses, and, consequently, limits the supply of men of education and competence, as it must increase the cost of professional aid when obtained; according to the expense of qualification must be the remuneration;—according to the distance of the practitioner, must be the charge for his journies and attendance.

"To the public it must also be a *calamity*, if it unnecessarily interferes with *early* and *competent* relief, under the casualties which flesh is heir to. But is there any real necessity for increased attendance previously to examination, and for increased fees for the diploma when obtained?—ninety-nine out of an hundred of the present diplomatists of the Royal College, were only required to attend six months; and yet the Court of Examiners, under their hands and college seal, certify, that such members 'have been deliberately examined, and found fit and capable to exercise the art and science of surgery;' and they are authorised to practise the said art accordingly. Let it then be asked, are the three thousand one hundred and ninety* members of the Royal College now practising the art and science of surgery in London, or various parts of the world, under the authority of the said College, competent or not competent to what they undertake? If they are competent, can there be even a shadow of justice, or of reason, for making the expense of a diploma (when it is to be no longer optional, but a *sine-qua-non* for practice) nearly double?—unless, indeed, the Royal College means to say to Parliament that the human intellect has become so deteriorated,—that the capability of acquiring knowledge has so lessened, that double the attendance is now requisite for former acquirements, and to make a qualified practitioner.

"After every consideration of the subject, then, it does appear that the Royal College must have authorised their present diplomatists to practise, when their studies had been insufficient, and they were *not* 'fit and capable;'—that *energy* and *talent* with hospital students are no more;—or, that they have doubled the attendance for an examination for their diploma without any sort of necessity, while such increased attendance and expenses must be burthensome to most pupils, *ruinous* to some, and detrimental to the best interests of the public,—whose actual wants require, that the facility by which they may be supplied with educated and competent aid should be rather increased than diminished.

"The circumstance, also, of excluding from the practice of surgery and midwifery all those students whose pecuniary means may be unequal to the recent arrangement for obtaining the College diploma, and the check it must give to parents, who will fear to bring up their sons to a profession made so expensive and uncertain, must be deserving the consideration of government; as it may affect the first interests of the nation, while a possibility exists, at some future time, of fresh commotions in foreign politics, which may suddenly call for a large supply of professional aid for the army, navy, and colonies.†

"* The numbers of the diplomatists of the Royal College, according to the list officially circulated from Lincoln's Inn Fields during the present year."

"† The difficulty of obtaining sufficient surgical assistance after the expedition to the Helder in 1794, and at the commencement of the late war, must be yet within the recollection of some of his Majesty's ministers.

"As

"As to the *third* head of inquiry, it cannot be doubted that many students must be unable to comply with new regulations, so greatly adding to, and, in some respects, nearly doubling the cost of an already highly expensive education, who may have acquired much insight into their professional duties; and, after a winter's attendance upon an hospital and lectures (which at best may be only managed for them but with considerable difficulty on the part of their friends) be equal to give proof of competence, and become useful servants of the public; while it may be utterly impossible for them to have a year's residence in town, to pay a year's hospital fees, leading to a further fee of thirty-two pounds ten shillings, or twenty-two pounds for a diploma (now unnecessary); and thus their legitimate prospects,—the best hopes of themselves, and of their families, be sacrificed.

"Should the Royal College substantiate their position, that increased attendance and expenses are *now*, in general, found necessary for their diploma, it cannot be expected that *all* must want double opportunities of acquiring science for competency. Is, then, the assiduous, studious, and well-informed pupil to suffer—to be excluded from a profession in which he must be useful, and to which he may be an ornament, because some of his brother students, idle, dissolute, or without talent, require double the time and pecuniary expenditure for equal sufficiency.

"The second clause enacts, 'That every person who shall have been so examined, and shall have received such diploma or testimonial, under the seal of one of the said three Royal Colleges, shall be entitled, and shall have the right to practise surgery in any and every part of his Majesty's dominions, any law or custom to the contrary notwithstanding.'

"The third clause provides, 'That all and every person, or persons, who shall have been duly examined, and shall have obtained a testimonial or qualification as a principal surgeon in his Majesty's army or navy, and who shall have actually served in that capacity, shall be entitled to practise surgery in any and every part of his Majesty's dominions.'

"The propriety and justness of this clause cannot be doubted.

"The fourth clause says, 'Whereas, by a certain statute of the Parliament of Ireland, passed in the thirty-sixth year of the reign of his present Majesty, intituled, 'An Act for the further Regulation of Public Infirmaries or Hospitals,' it is enacted, That from and after the passing of that Act, no person should be capable of being elected surgeon to a county Infirmary or Hospital, who should not previously have obtained letters testimonial of his qualification, under the seal of the Royal College of Surgeons in Ireland; and that no other qualification or examination should be necessary to make any person capable of being elected surgeon to such Infirmary or Hospital: And whereas, by another Act, passed in the fifty-fourth year of the reign of his present Majesty, intituled, an 'Act to amend several Acts for erecting or establishing public Infirmaries or Hospitals in Ireland, so far as relates to the Surgeons or or Apothecaries of such Infirmaries or Hospitals,' it is provided, that letters testimonial of the College of Surgeons in Ireland, shall be laid before the grand juries in the said Act mentioned previous to the requiring or making any presentment of any sum of money to be surgeon of any Infirmary or Hospital by such grand juries: And whereas, it is just and expedient that the provisions of the said Acts should be extended to the members of the Royal College of Surgeons in London and in Edinburgh; be it therefore enacted, That the Members of the Royal College of Surgeons in London, and of the Royal College of Surgeons in Edinburgh, shall be eligible to all the offices and appointments mentioned in the first recited Act, and shall be entitled to all benefits and advantages given and intended by the second recited Act, on the production of the diploma or testimonials, under the seal of their respective Royal Colleges, in the same manner as the members of the Royal College of Surgeons in Ireland have been, since the passing of the said recited Acts.'

"In an highly interesting essay, on 'the Present State of the Medical Profession,' it is observed, that, in Ireland, 'surgeons are restricted to *surgery* only, and interdicted from the practice of *pharmacy*, under the penalty of the forfeiture of their rights.'* There may, therefore, be considerable difficulty in making any *law* on the subject of surgery and midwifery, applicable to both countries; as in England, the state of society requires, and custom recognizes, a *general practitioner*."

The following is the clause relative to the practice of midwifery.

"Whereas surgical aid is frequently required in midwifery, and it is expedient that male persons so practising, should be qualified to render such aid, be it therefore enacted, that from and after it shall not be lawful for any male person to practise midwifery, unless he shall have obtained a diploma or testimonial to practise surgery under the seal of one of the said three Royal Colleges; or unless he shall have obtained a testimonial or qualification as a principal surgeon in the army or navy, and shall have actually served in that capacity."

Nothing can be more painful than the appearance of any occult design, in whatever relates to the practice of our art; and, if there is a part in which we ought most to avoid it, that part is midwifery,—an art in which every female will have her opinion, and, in which, if she finds herself treated with the insolence of secresy, she has a right to arm herself with suspicion on every side. Let us see what the preamble of the above clause would lead to: "Whereas surgical aid is frequently required in midwifery," &c. What would be

"* Kerrison's Inquiry into the Medical Profession, p. 50."

the the inference? The answer is plain—therefore, besides a knowledge of midwifery, those who are to be consulted on difficult cases should have an accurate knowledge of the anatomy of certain parts; and no person is fit to examine practitioners for midwifery without such a knowledge. Be it therefore enacted, that when the legislature shall appoint such examiners in midwifery, they shall be themselves examined touching their own qualifications, by competent persons, members of the said College of Surgeons. Perhaps our readers may laugh at this. So do we. But such amusement is innocent compared with the trick of gaining the profits of examination for midwifery, not because those who expect it are competent midwives, but because thay understand one branch of science which is frequently necessary in that practice. No, we beg pardon, it is not the examination for midwifery, but an exemption from any such examination, provided they have a diploma in surgery, or, which is still better, provided they have served in the army or navy.

On these subjects we are open to conviction. What we have offered arises from the perusal of the tract before us: we shall be glad to see any arguments on the other side.

---

*Medico-Chirurgical Transactions, published by the Medical and Chirurgical Society of London.* Vol. VII. Part I.—Longman and Co. 1816.

*(Concluded from p. 78 of our last Number.)*

*History of a Case of Chorea Sancti Viti, occurring in an Adult, and cured in an unusual Manner.* By Mr. Kinder Wood, Member of the Royal College of Surgeons in London, and Surgeon in Oldham.

This is a very interesting, and (as the patient recovered,) not less entertaining, history. The disease was brought on, as far as we can judge, by long suckling, and, in our opinion, the cessation from that drain has hardly credit enough given it in the history of that cure. We shall not, however, enter more minutely into a case on which various opinions will be formed, but conclude with admitting that the history is worthy of recording, and of the space which it occupies in these memoirs.

*Particulars concerning the Structure of a Monstrous Fœtus;* by Mr. Maunoir, Professor of Surgery at Geneva.

Probably this monster may be different from any other on record. We are not disposed to hunt for authorities to confirm or refute such a position.

*Observa-*

*Observations on a Change of Colour in the Skin, produced by the internal Use of the Nitrate of Silver;* by J. A. Albers, M.D. of Bremen.

Several instances of the above are produced, all well confirmed. The fact is highly interesting, and there is no reason to doubt the truth of any of the narrators. We shall not enter into any chemical inquiry into the cause, but content ourselves with remarking that we have not found sufficient reason to impute any great virtues to the remedy, and can, therefore, dispense with it without reluctance.

Such are the medical papers contained in this collection; the chirurgical, as we remarked, consist principally of bold operations, deserving of record, though without any important advances in the pathology of surgery.

*Case of Un-united Fracture of the Os Humeri, treated successfully by the Seton;* by Josias Stansfield, Esq. Surgeon to the Leeds Infirmary.

This is a very great improvement in the mode of treating such a condition of the bone beyond the old practice of cutting down and rasping the broken ends.

*History of a Case of Wound in the Face, requiring the Operation of Tying the Common Carotid Artery, which was performed with success;* by Charles Collier, Esq. Surgeon to the Forces.

A case well worth recording, but which it cannot be necessary to describe.

*History of a Tumor successfully removed from the Face and Neck, by previously Tying the Carotid Artery;* by William Goodlad, Esq. Member of the Royal College of Surgeons, and Surgeon at Bury, Lancashire.

This case is still more interesting.

The tumour extended "from near the external canthus of the left eye, down the cheek to the infra-orbitary foramen and the root of the ear, which was elevated by the tumour, and, passing under it, it extended behind the mastoid process. Anteriorly, it extended to the chin, and to the trachea, which it partially covered, and hung pendulous over the clavicle. The circumference of the base of the tumour, when last measured, was twenty inches, since which period it had increased rapidly, but I regret not having ascertained its exact size; it must, however, from the space it occupied, have been at least twenty-eight inches.

"In the upper portion, comprised between the cornu of the os hyoides upwards to above the zygoma; the tumour in the base was as large as, or larger than in its middle or apex, but below this point, point, its attachment to the neck was less extensive; and, though hanging pendulous over the clavicle, its lowest connection with the neck, was three-quarters of an inch distant from that bone. On elevating the tumour in this lower space with a hand on each side, and passing the fingers at the same time under it, it might clearly be ascertained that there was no connection with the vessels: but, as it hung over the trachea, and was connected with it, it required a very careful examination to be convinced that they were not united. The cornu of the os hyoides was, however, moveable under the tumour, and independent of it; respiration was tolerably free, when in an erect posture, and, on passing the fingers forcibly between these parts, I was pretty well convinced that they might be separated. The œsophagus here was too deep to be implicated in the disease.

"Above the cornu of the hyoid bone, the base of the tumour was very extensive, and deep, impeding deglutition considerably. On directing the finger, passed into the mouth, towards the base of the ramus and angle of the maxillary bone, and into the fauces; the intervening substance appeared considerable, and authorized an opinion that the fauces would not be cut into, nor indeed exposed, by its removal. The submaxillary gland was pressed inwards, but did not appear enlarged or hardened, and the membrane of the cheek was also healthy, though the tumour appeared in contact with it.

"The disease commenced behind the angle of the jaw, and now extended beyond the mastoid process, and was so closely connected with the subjacent parts, that the finger could not be passed under it. Its connections here, therefore, were only to be ascertained by collateral circumstances; and, as the whole site of the parotid gland was covered by it, it became a question worthy of serious consideration, how far that substance was enveloped in the disease, particularly as it had been stated on most respectable authority, that its removal was impracticable. The patient's being able to open the mouth and masticate, was a complete proof, that if the whole substance of the gland were diseased, it had become dragged from its situation by the weight of the tumour, and that the tumour itself did not dip into the fossa behind it. Yet every other circumstance rendered it certain that the parotid gland was enveloped in the disease, which was indeed verified in the operation. But the extent of the disease here became of less importance if the carotid artery were previously secured, as I intended. I ought to have observed, that the tumour was perfectly moveable, though its motions were very limited, and that it had, therefore, no bony attachment either to the jaw or to the zygoma.

"The surface of the tumour was divided into large tuberculi, and the apex of each tubercle was rendered more prominent by a collection of fluid: it was fleshy, but had neither the hardness nor any other external character of carcinoma. There was no absorbent gland affected in its neighbourhood, and, though ulcerated in two points, one of which was extensive, the aspect of the ulcer was not forbidding, being partially granulating and partly sloughing. Fungus hæmatodes was, therefore, not to be dreaded. Yet a discouraging circumstance arose from a charlatan's having, as he believed, succeeded in removing the complaint by the knife, at an early period of its history, on which occasion the hæmorrhage was very alarming. After a short time, the disease reappeared, and had only been nine months in attaining its present enormous size. Large varicose veins spread over the surface, and as the skin was uniformly diseased, ulceration might be expected to extend to them, and hæmorrhage to be an inevitable consequence."

The great objection to the operation was the danger of hæmorrhage, to prevent which, no certain remedy occurred but previously securing the carotid artery, which it was expected would also prevent any interruption to the operation from wounding the smaller blood-vessels. We shall not detain the reader by a description of the first operation, that of tying the artery, because, we presume, any surgeon hereafter, who wishes to repeat it, will consult this and all the former accounts. It is, however, well described, and under many difficulties proved successful.

The tumour being of the incysted kind was more easily managed, the greater part being detached by the fingers only. When the knife was necessary, a considerable quantity of venous blood flowed, (we have often suspected that these tumours are fed chiefly by veins,) but very few ligatures were required. The wound was more readily healed than could have been expected, considering its extensive surface, and that sufficient healthy skin could not be preserved to cover the whole.

*History of a Case of Aneurism of the Femoral Artery, for which the Operation of Tying the External Iliac Artery was performed;* by CHARLES COLLIER, Esq. Surgeon to the Forces.

This aneurism was the effect of a gun-shot wound, which had penetrated as far as, and seemed to remain in contact with, the artery for more than two months. The integuments had healed before the case was suspected. Soon after the operation, all the symptoms that might be apprehended from the deprivation of blood appeared in the limb, and the patient died on the third day after. The following is the account of the dissection.

"On opening the abdomen in the usual manner, there appeared a general flush of the intestines; the reflected peritoneum was of its usual transparency, and the cœcum was adhering to it by lymph over the iliac muscle. The ligature was on the artery close to Poupart's ligament, about an inch below the giving off the circumflex, and some quarter of an inch from giving off the epigastric. The

The wound of the operation was made as I intended. There was a small communication between the femoral artery and vein at the side of the tumour, about an inch and a half below the origin of the profunda; on the superior part the covering of the aneurism was formed by the sheath of the vessels, and the fascia of the thigh. The profunda was neither thickened nor enlarged. The whole limb was in a state of gangrene."

*A Case of Ossification and Bony Growth of the Cartilages of the Larynx, preventing Deglutition;* by FRANCIS TRAVERS, M.D. of Newark, Nottinghamshire.

This case is well worth recording, which is done with equal judgment and modesty, but no practical inferences can be drawn from it.

*Case of Gun-shot Wound of the Shoulder Joint, where the Head of the Os Humeri, together with Parts of the Humerus, were successfully removed;* by WILLIAM RICHARD MOREL, Esq. Surgeon to the Forces; Surgeon to the York Hospital, Chelsea; and Surgeon to the Westminster Hospital.

*Observations on the Treatment of Varicose Veins of the Legs;* by B. C. BRODIE, Esq. F. R. S. Assistant-surgeon to St. George's Hospital, and Lecturer on Surgery.

The improved treatment here proposed, and which the author has found successful in several cases, four of which are related, consists in making a small incision in the integuments, cutting through the varix, and then bringing the segments of the skin together by adhesive straps. This certainly seems an improvement on the ligature, the ill effects we have had occasion to record in a former number. Mr. Brodie concludes his paper with the following remarks.

"From the result of the foregoing and of many other cases, I am induced to conclude, that the operation which has been described, may be frequently employed with great advantage to the patient. At the same time I wish to be understood as recommending the adoption of it, not indiscriminately, but with a due attention to the circumstances of each individual case. The cases for which it is fitted, are, not those in which the veins of the leg generally are varicose, or in which the patient has little or no inconvenience from the complaint, but those in which there is considerable pain referred to a particular varix, or in which hæmorrhage is liable to take place from the giving way of the dilated vessels, or in which they occasion an irritable and obstinate varicose ulcer."

We perfectly agree with the author, that if the varices are unattended with pain, we should neither propose this nor any other operation.

The second part of this volume is now published, and shall be noticed in an early Number.

 *Practical*

*Practical View of the principal Chirurgical Operations, founded on his own experience*; by C. KLEIN, M.D. Physician to H. M. the King of Wurtemburg. Fascic. I. with 18 engravings. 4to. pp. 80. 1815.

THIS work may be considered as one of the most valuable enrichments surgery received during the last destructive war. In the year 1814, the author had the direction of three field hospitals, in which were above 2500 Russians, among whom were about 1900 dangerously wounded: he communicates, in this fasciculus, the results of the amputations, which were here undertaken under such favourable circumstances, that only 10 died of 109 that suffered amputation. The amputation of the arm from the shoulder-joint was performed by him six times with the best success.

